# Supplementary material for: No significant association of repeated messages with changes in health compliance in the COVID-19 pandemic: a registered report on the extended parallel process model
Source: PeerJ. 2021 Jun 3;9:e11559. doi: 10.7717/peerj.11559 (PMC8180189; doi:10.7717/peerj.11559)
Supplement: Supplemental Information 2 [file peerj-09-11559-s002.docx]

| 自己効力感  (1-全く同意しない, 7-とても強く同意する) | |
| --- | --- |
| _1_ | 新型コロナウイルスの感染を予防するために，私は下線部の感染予防対策を実施する能力がある。 |
| _2_ | 新型コロナウイルスを予防するために，下線部の感染予防対策をすることは易い。 |
| _3_ | 新型コロナウイルスを予防するために，私は下線部の感染予防対策を実施することができる。 |
| 反応効力感  (1-全く同意しない, 7-とても強く同意する) | |
| _4_ | 下線部の感染予防対策を実施すると，新型コロナウイルスの感染を予防できる。 |
| _5_ | 下線部の感染予防対策を実施することは，新型コロナウイルスの感染を予防する効果がある。 |
| _6_ | 下線部の感染予防対策を実施することは，新型コロナウイルスを撃退するのに有効である。 |
| 易罹患性認知  (1-全く同意しない, 7-とても強く同意する) | |
| _7_ | 自分は新型コロナウイルスに感染する危険性がある。 |
| _8_ | 自分は新型コロナウイルスに感染しうる。 |
| _9_ | 私は新型コロナウイルスに感染しやすい。 |
| 重大性認知  (1-全く同意しない, 7-とても強く同意する) | |
| _10_ | 新型コロナウイルスは重大な脅威である。 |
| _11_ | 新型コロナウイルスは有害である。 |
| _12_ | 新型コロナウイルスは深刻な脅威である。 |
| 行動意図  (1-全く同意しない, 7-とても強く同意する) | |
| _13_ | 今後，アルコール消毒液で手を消毒する時，ポンプ部分を下までゆっくり押して，たっぷり手に取って使う。 |
